# Obstetrics and Diseases of Women and Children

**Published:** 1861-03

**Authors:** Wm. B. Atkinson

**Affiliations:** Lecturer on Obstetrics, and Diseases of Women and Children


### OBSTETRICS, AND DISEASES OF WOMEN AND CHILDREN.

BY WM. B. ATKINSON, M.D.,

LECTURER ON OBSTETRICS, AND DISEASES OF WOMEN AND CHILDREN.

### I.—*Placenta Prœvia.* By T. Gaillard Thomas, M.D., of New York.

After a careful resumé of the history, varieties, mortality, etc. of the subject under consideration, Dr. Thomas speaks of the "indications for treatment." These are to deliver the child as soon as possible, and thus prevent the necessity for *gradual* dilatation of the os and cervix; or to alter the state of affairs at the cervix so that gradual dilatation may go on without producing hemorrhage.

The methods of treatment are version; partial separation of the placenta; complete separation of the placenta; rupture of the membranes; the tampon, if forced to wait.

He supposes two cases, the first of a grave character. Here, where the hemorrhage is serious, delivery must be accomplished as soon as possible. If the patient's strength be sufficient to bear the operation, the child be living, and the os uteri dilatable, turn without delay.

If the exhausted state of the patient, or the death of the child render the operation too hazardous, or in the latter case useless for the safety of the child, a portion or the whole of the placenta may be separated; a part, if sufficient, the whole otherwise.

If a rigid os prevents turning, and the child be living, if the woman could bear a further loss, it would be well to await the time when dilatation will occur before separating the placenta, and thus destroying the fœtus. While waiting, danger may be avoided, and the process hastened by partially separating the placenta and placing a sponge saturated with a solution of perchloride of iron against the os, and keeping it there by filling the vagina with a tampon, which, in itself, accomplishes both objects. Better still, the colpeurynter might be used, or a bladder tied to the end of a self-supplying syringe, introduced into the vagina in a collapsed state, and then filled with water. This, however, is but temporizing, and it is seen that the principles of treatment are narrowed down to immediate delivery; waiting until the state of the parts will allow it; or altering the state of affairs at the outlet of the womb so that the natural expulsion of the child may occur without danger.

In certain cases it is necessary to decide between complete separation of the placenta and version.

*The latter is preferable—*

When the child is living; at full time; when the patient's strength is good; when the soft parts are dilatable; when the pelvis is not deformed; in multiparæ.

*The former is preferable—*

When the child is dead; before full term; when the patient is exhausted; when the soft parts are rigid; when the pelvis is deformed; in primiparæ; during an epidemic of puerperal fever.

It has been objected to separation of the placenta in cases where the soft parts are rigid, that if the hand can be introduced for that procedure, version would be practicable. But, in certain cases, two fingers will suffice to detach the placenta, and even if it required the whole hand it would be much less dangerous to stretch a doubtful os for its admission to the wrist, than for the introduction of the entire forearm and subsequent extraction of the child.

Few points in obstetrics require a greater amount of judgment in their decision than the period at which version should be performed in placenta prævia; for on the one hand, if it be performed too soon, a laceration of the unyielding os may occur, and the woman be exposed to the great risks of post-partum loss from the immensely developed vessels at the placental site, as well as to those imminent ones of phlebitis; while on the other, if too long delayed, her forces will become so exhausted that the shock of the operation will produce death.

It is a very prevalent error that, whenever version is called for by the loss of blood, this loss will itself render the parts dilatable.

He next supposes a case unaccompanied by grave symptoms. When the hemorrhage is slight, the obstetrician may resort to minor means at first. Thus, if the placenta is placed laterally, the membranes may be ruptured in the hope that pressure from the presenting part may check the flow. Should this fail, we may employ Barnes's method of detaching by the finger, only that part of the placenta attached to the cervix, and leaving that attached above this point still adherent. Thus is accomplished at once the separation of all that must necessarily occur to permit the passage of the child; succeeding uterine efforts do not affect that portion attached to the body; and tonic contraction of the uterus closes the open vessels of that organ, while coagula do the same for those of the placenta. After which, should the flow continue so as to require aid, version should be resorted to under the restrictions just mentioned; or if the head is within reach of the forceps, they should be employed. If it be impossible to introduce the fingers into the os for the performance of Barnes's method, the vagina should be plugged until the parts are in the proper condition.

Should the placenta be directly in front so as to preclude the determination of the position of the child by the touch, endeavor to ascertain by palpation and auscultation. If forced to turn without knowing the position, always employ the *left hand*, as the occiput is much more commonly to the left acetabulum, and that hand would be applicable to such a case. Do not waste time in breaking through the placenta, but pass the hand between it and the uterus. He sums up as follows: 1st. Os dilatable and woman not exhausted, deliver immediately by version or the forceps. 2d. Os dilatable and woman exhausted, detach a part or the whole of the placenta; apply styptics, and stimulate. 3d. Os rigid and woman not exhausted, detach the portion of the placenta nearest the cervix, and, if necessary, apply styptics and tampon, or colpeurynter. 4th. Os rigid and woman exhausted, detach part or whole of the placenta, apply sponge saturated with perchloride of iron, and support strength by stimulants.—(*Amer. Med. Times*, Jan. 19, 1861.)

II.—*A New Sign of Post Partum Detachment of the Placenta.* By JOHN CLAY, M.R.C.S., of Birmingham.

From investigations, with a view of improving upon the old plan of management of the delivery of the placenta, Dr. Clay ascertained that a very simple sign existed, by which its separation, after the birth of the child, might be indicated, having tested it in upwards of nine hundred cases. Before dividing the umbilical cord he applies two ligatures. If the maternal part is now examined, it will be found in a flaccid condition, and almost free from blood; but after an interval of from one to three minutes, it will be found to have acquired specific weight, and that the vessels are more or less filled with blood. The one fact may be ascertained by poising the cord on the fingers; the other by slightly grasping the cord near the vagina, with the thumb and forefinger of the left hand, and, with the right hand, suddenly compressing it, when a well-marked sense of fluctuation is perceived, a kind of resilience like that felt when an elastic tube filled with fluid is suddenly compressed. When the placenta is detached, the cord loses its increased specific weight and the hydrostatic property just mentioned. This is so invariable, that *the loss of the previously acquired hydrostatic properties of the cord after the birth of the child constitutes the sign of detachment.*

The whole of the phenomena are manifested in three stages—flaccidity, repletion, flaccidity.

If the cord be tightly grasped by a spasm of the os, or by irregular contractions of the uterus, the *loss* of the hydrostatic properties may for a short time be delayed. These signs are not, of course, equally marked in every case. When the uterus is flaccid, they are but slightly manifested, though perfectly reliable. When, on the other hand, the contraction is firm, the most inexperienced may detect them. In cases of partially adherent placenta, the disappearance of the hydrostatic properties, and the failure to deliver the placenta by the usual manipulations, indicate the necessity for artificial detachment by introducing the hand. In twin cases, the signs persist till after the birth of the second child, except where two placentæ are present.

It sometimes occurs that the placenta is separated simultaneously with the birth of the child. Here the first series of phenomena are absent, and it may be prudent to wait before proceeding to extract the placenta, although it may be generally effected with safety.

The practical value of these facts is obvious, as the placenta, when thus known to be separated, may be at once extracted. The prompt delivery of the placenta is very important, as the uterus then contracts more efficiently, the risk of hemorrhage is not so great, and it may be fairly assumed that the convalescence is less protracted.

To inexperienced practitioners it might be a safe instruction to impart, not to interfere in the extraction of the placenta so long as the hydrostatic properties herein defined are persistent.—(*Dub. Quar. Jour.*, November, 1860.)

III.—*Sub-Involution of the Uterus after Delivery.* By J. Y. Simpson, M.D., of Edinburgh.

After relating two cases of the affection, Dr. Simpson spoke of its pathology. In these cases, the retrograde metamorphosis of the uterus, which is natural after labor, has not taken place, or so imperfectly that it has but slightly diminished in size.

There is here a hypertrophy which is pathological only in its permanency. This is generally the result of rising too soon after confinement, repeated miscarriages, or metritis. Its symptoms are a feeling of discomfort in the abdomen, a bearing-down, distress in connection with the bladder and rectum, pain in the lower part of the back, sometimes numbness of the lower limbs. The menses do not return regularly, or are profuse and painful, and leucorrhœa to a greater or less extent is present.

The diagnosis is made out by passing the hand over the abdomen, when the enlarged uterus is felt like a tumor rising out of the pelvic cavity, and by a vaginal examination, the cervix is found to be enlarged, and the whole organ unusually large and heavy. It is of importance here to examine with *both* hands simultaneously, the forefinger of one hand exploring by the vagina, while those of the other are applied to the anterior abdominal wall. With the uterus thus between the hands, the hypertrophy is easily recognized, and the absence of fibroid masses in the walls is known by the regularity in shape of the organ. But as there might yet be a fibroid tumor in the submucous layers of the uterine walls, the sound must be introduced. This should penetrate two and a half inches; but when the uterus is enlarged it may enter much farther. If a tumor be present, an obstruction is met with, which may require some dexterity to pass. As the great weight of the organ, aided by inflammatory adhesions, may cause displacement, as retroflexion, etc., these must be expected; and when an obstruction occurs, the sound may be passed by turning its concavity backward, when it will enter deeper if a retroflexion is present.

The affection may be detected within a few weeks or months after delivery. These are the acute types, but, generally, many months or even years may elapse before the physician is consulted. It will vary in degree from a slight hypertrophy to the normal state of a fully pregnant womb.

In the acute form it should be treated by local antiphlogistics, which cause absorption to set up. If the patient is not debilitated, leeches should be applied to the vaginal portion of the uterus, the perineum, or circle of the anus. In chronic cases, counter-irritants must be applied to the surface of the sacrum or abdomen, as antimonial or croton ointments, tincture of iodine, or blisters. A series of small blistering-plasters may be applied over the lower part of the abdomen, as one every second or third day. At the same time the vaginal portion of the cervix should be kept immersed in ointments of mercury, or iodide of lead, or bromide of potassium, as by means of medicated pessaries.

When these are not sufficient to effect a cure, deobstruent remedies may be given internally; the most efficacious are iodide or bromide of potassium. The latter has the advantage that it may be kept up for a long time without any bad effects, and is also an excellent tonic. The dose should be large, as six, eight, or ten grains three times a day. When the patient is much debilitated, it is best to add iron, manganese, etc. Some cases are very obstinate, and require a long continuance of the treatment. If, however, the uterus could be induced to *increase* in growth, it may be confidently hoped that this will be followed by a decrease and the return of the organ to its normal dimensions. This transient increase may be produced by the introduction of a sponge tent, etc. Then, having caused the uterus to take on hypertrophic action, by actively following it up by the remedies as above, a cure is effected. In some obstinate instances, Dr. Simpson has been obliged to repeat, from time to time, this process of artificial irritation and dilatation before a perfect cure resulted.—(*Med. Times and Gaz.*, Jan. 5, 1861.)

IV.—*Glycerin and Camphor in Suppression of the Secretion of Milk.* By DR. JURIAH HARRISS, of Savannah, Georgia.

Having carefully tried belladonna in cases where the suppression of the lacteal secretion became necessary, Dr. Harriss has arrived at the conclusion that it too often is without profit to the patient. He usually prescribes a saturated solution of camphor in glycerin, to be gently applied over the surface of the mammæ with a flannel cloth; this to be renewed several times a day.

Camphor is freely soluble in glycerin, and by the combination the effect of the former is obtained, while the latter protects the breast from the air. In sore nipples also, this has proved very useful, though he generally adds four grains of tannin to an ounce of the mixture.

The next best remedy to arrest threatened abscess, is the application of adhesive plaster over the whole gland, fitting it closely and leaving a hole for the nipple. We thus compress and support the gland, and officious meddling by friction is prevented and rest secured.—(*Savannah Jour. of Med.*, Nov. 1860.)

V.—*The Pessary a Hurtful Remedial Agent.* By DR. W. M. TURNER, of Petersburg, Virginia.

The only cases in which Dr. Turner regards the pessary as proper, are those in which *complete* prolapsus has occurred, the ligaments being so lax as to permit the uterus to protrude through the genital fissures. Even here, he regards it as exchanging vaginitis for metritis. His objections to this instrument are that it presses on the vagina and occasions inflammation and ulceration, often resulting in fistulæ; it is unsound curative practice to treat effect instead of cause; or rather it is far better to give tone to the ligaments of the uterus and vagina; the inconvenience arising from the adjustment of the pessary, getting the particular size, cleansing it, and the disgust it produces in the mind of a delicate woman. He seems to prefer suspensory bandages, astringent injections into the vagina, and absolute, continued rest in the horizontal position, with the pelvis elevated. He has found, as a substitute for the pessary, sponge of a cylindrical form, about four inches in length, to act very well. This is saturated in a strong decoction of red-oak bark and placed in the vagina by means of the speculum. This acts as a pessary in a measure, and does not press on any particular point of the vagina, and is an excellent means of applying a local astringent. It should be removed daily and cleansed.

His treatment for all malpositions of the womb is the use of tonics, rest, astringent injections and a proper suspensory bandage. The latter may be made of calico or muslin, and should be of a triangular shape, two sides of the triangle being curved to fit the abdomen; to the angles, tapes are attached. He always suggests, when patients are walking about, that the hoop skirt should be supported by straps over the shoulders, so that the superincumbent mass of clothing may not drag around the waist.—(*Amer. Med. Times*, Dec. 22, 1860.)

VI.—*The Use of Pessaries.* By Dr. P. Stewart, of Peekskill, New York.

After repeated trials of these instruments, Dr. Stewart has found so many inconveniences resulting from them, that he has abandoned their use and resorted to the direct application of astringents by the syringe or sponge, the horizontal position, riding in an easy carriage, tonics and the suspensory bandage. He uses the sponge not as a support, but to carry up the astringent and keep it in contact with the relaxed tissues. He regards the bandage as a *sine qua non.* It is made by fitting nicely over the pubes a pad of thin sole leather, from eight to ten inches long, and three or four wide; a soft pad is placed over the spine, and both are furnished with loops. Two strips of saddler's webbing pass round the hips through these loops, one above the other, and fastened in front; the lower one being a little the shortest, that the pressure may be exerted from below. Another strap of rolled cotton passes round the head of each thigh, and fastened to the other pad by a small ring and an elastic band. His success since the abandoning of pessaries has been decidedly marked.—(*Amer. Med. Times*, Jan. 12, 1861.)

VII.—*An Inquiry into the Correctness of the Doctrines of William Hunter in regard to Retroversion or Retroflexion of the Gravid Uterus.* By Dr. W. Tyler Smith, of London.

Dr. Smith's attention was especially directed to the subject by the case of a lady who suffered from complete retroversion in the unimpregnated state. She became pregnant, and went the full time. What was the condition of the uterus here after impregnation? To answer this he carefully watched the progress of gestation in a number of such cases, and was "convinced that the most common cause of retroversion of the gravid uterus is not to be found in the state of the pelvis or bladder, but in the occurrence of impregnation in the retroverted uterus and in the tendency of the organ thus impregnated to develop itself in this position." The os uteri approaches the pubis, and the fundus projects toward the hollow of the sacrum. At length the bladder and rectum are so pressed upon as to interfere with their functions. Owing to retention of the uterus within the pelvis, there is little or no increase in the size of the abdomen. Abortion frequently ensues from the mechanical irritation of the uterus.

The great design will be to prevent the full retroversion or strangulation of the uterus in the pelvis. As long as retroversion was supposed to occur suddenly and mysteriously, nothing could be done to avert it; but if it dates from the beginning of pregnancy, we may do much by position and attention to the bowels and bladder, and also when it becomes necessary, give prompt mechanical assistance by passing the hand into the vagina and carrying the fundus above the brim. We ought then to endeavor to prevent a return of the retroversion. The fœtus acts as an intra-uterine pessary, the organ is strengthened, and we have a better chance of curing the affection than under other circumstances. The abdominal bandage should not be too tight; the patient should lie on her side, inclining to the prone position, but avoiding recumbency. The bladder and rectum should be kept empty. She should remain in bed longer than usual, and on getting up, if any tendency to a return exists, an air pessary should be worn.

*Dr. Oldham* agreed perfectly in these views. Such cases were common in hospital practice. He did not like the use of the air pessary, as it might do harm, and in the greater number of cases mechanical attempts at replacement are unnecessary. When expedient to interfere, he gently opened the orifice of the vagina so as to fill the canal with air. The vagina thus became much distended, and gentle pressure with the finger upon the fundus sufficed to push the organ up into the normal position.

*Dr. Barnes* had one year ago published similar views. But was sure that the affection also occurs from accident, and suddenly takes place. He had tried Dr. Oldham's plan in two cases, but failed.

*Dr. Waller* took the same ground with Dr. Smith. His rule was to do nothing in the majority of cases, unless the rectum and bladder required attention. He had withdrawn thirteen pints of urine in one case which had been mistaken for dropsy, and the patient made a favorable recovery.

*Dr. Priestley* said there were other dangers in the early months, as abortion at the third or fourth, and these ought to be prevented. There is often no difficulty in replacing the uterus; but when the patient gets up it falls back again. —(*Brit. Med. Jour.*, Nov. 17, 1860.)

VIII.—*The Pathological Lesion of Phlegmasia Dolens.* By W. TILBURY FOX, M.D., of London.

This paper was an appendix to a former, and its object was to solve two main questions: 1. The essential condition or lesion of the limb in this affection. 2. The mode of production of this lesion.

In regard to the first, it was maintained that the presence of fibrinous serosity in, with more or less hypertrophy of, the fibro-cellular tissue, is the essential, the sufficient, pathological lesion; that the inflammation, the abscess, the sloughing, etc. are not peculiar or necessary parts of the affection, but common to many diseases, and the result of an eliminative act to rid the system of some *blood-poison;* that the latter may produce lymphatic and venous obstruction, and hence phlegmasia dolens; but it passes beyond this, giving rise to a distinct disease, such as abscess, pyæmia, etc.

Phlegmasia dolens in these cases is a local complication of the general disease, and the general symptoms are but parts of the phlegmasia. There are then two types of this disease:—

*a.* The complicated, in which an eliminative process (inflammation, abscess, etc.) takes place, these being the answer acts of the tissue to the blood state; the epidemic form, in which an effect is produced by a virus, superadded to what occurs in

*b.* The other class—the uncomplicated—where the blood state does not require any eliminative actions to be performed on the part of the tissues, but where simple obstruction exists, such as pressure by tumor, etc., and simple thrombus; this being the essential, simple, ample disease, all else being superadded and accidental.

It having been shown that venous obstructions produced œdema *only*, and this *plus* lymphatic obstruction, (phlegmasia dolens,) the second question as to the mode of production of the lesion in the limb might be stated thus: How can obliteration of the lymphatics produce the peculiar change of the limb in phlegmasia dolens? The lymphatics being obstructed, the three offices of removing waste, of absorption, and of formative power, could not come into play.

It was then argued at length that the amount of lymphatic distribution and fibro-cellular tissue are in direct ratio—that fibrin is the pabulum of the latter tissue—and that Virchow's views on this point clash with sound doctrine. Lymphatic obstruction being followed by the retention of fibrinous serosity in the cellular tissue, the inference allowed by the foregoing facts was, that *one* office of the lymphatics is to remove all superfluous material from the cellular tissue; to keep the balance of nutrition there correct; hypertrophy and retention of fibrin in the cellular tissue ensuing upon lymphatic obstruction: and this explanation is confirmed by the behavior of the lymphatics in cases of cancerous ulceration, etc. (When a blood-poison is present, *special tissue actions*—as abscess and the like—are superadded.) It was attempted to be shown that absorption by the lymphatics from ulcerated surfaces might give rise to thrombus at the entrance of the thoracic duct into the junction of the jugular and subclavian veins, and thus account for phlegmasia dolens occurring in the upper extremity in cases of disease of other parts of the body, *e.g.* cancer uteri.—(*Brit. Med. Jour.*, Oct. 20, 1860.)

IX.—*Rare Termination of a Fibrous Tumor of the Uterus.* By DR. LUMPE.

The patient, aged thirty-one, in 1848 had two miscarriages, after which she was subject to a profuse menorrhagia, and shortly perceived a tumor, accompanied with pains in the lower part of the abdomen. She became much emaciated, and in 1856 consulted Dr. Lumpe, and by the use of iron and quinia was much improved. In 1859 the hemorrhage was renewed, and at this time the tumor was about the size of an adult head, movable, situated near the fundus uteri, with little sensibility, no fluctuation, and of a uniform consistence. Œdema of the left leg now occurred, and, suddenly, jet-like discharges of a sero-purulent and fetid fluid took place, and which contained organic detritus. These continued for four months. The œdema then had almost disappeared, and spontaneously a diuresis and an abundant diarrhœa set in. A few weeks later, however, a general anasarca invaded the whole body, which, with extreme anæmia, anorexia, nausea, and sleeplessness, persisted two months. She then began to improve, the fetid discharges diminished, and by the end of another month the menstruation had become normal. During all this time the tumor was gradually diminishing in volume. A short time prior to the return of the menstruation, she had commenced to pass with the vaginal evacuations *calculous concretions* of various sizes, analogous to those sometimes found in the placenta. After the reappearance of the menses, these concretions still appeared in great numbers. She now rapidly improved, and by June, 1860, the body of the uterus, superadded to the tumor, was but little larger than the first.—(*Zeitschrift der Gesellschaft der Aerzte zu Wien*, No. 29, 1860; *Gaz. Hebdom.*, Nov. 1860.)

X.—*Contribution to the History of Abortions and Uterine Polypi.* By PROF. ROKITANSKY, of Vienna.

In making an autopsy of a young female, M. Rokitansky discovered in the uterus an ovule with extremely curious peculiarities. The cavity of the uterus was not notably dilated; the mucous membrane was swollen. The neck, on the contrary, was distended and of a globular form; its volume was almost double that of the body; it contained a rounded sac, reddish blue, fluctuating, and measuring two inches in diameter. This was suspended by a pedicle implanted on the anterior wall of the uterus, a little above the internal orifice. This pedicle was composed of hypertrophied mucous membrane. The uterine caducous coat had ruptured around the pedicle and formed at its junction with the ovule a species of ring. The ovule was composed of the normal elements. It was covered by the decidua reflexa, which presented inferiorly a circumscribed mortification extending equally into the whole thickness of the chorion. This was thin at its inferior part, but quite thick near the pedicle; it was ecchymosed throughout. The internal face was covered with a delicate amnion, which inclosed in its cavity an embryo an inch in length. The external surface of the ovule was surrounded by a very thin albuminous layer; it had not any connection with the internal face of the uterine neck, from which it was separated by clots of blood and a bloody liquid. The mucous membrane of the neck was much congested, reddish blue, rough, and covered with a whitish, thickened epithelium. The vagina contained also clots and a bloody liquid.

M. Rokitansky made a similar observation in another case, only the embryo had disappeared. He interprets these facts in this way:—

The egg has naturally contracted its adhesions to the mucous membrane which has furnished the normal covering. Then it has been displaced, and has descended into the cavity of the neck, where it has been developed. The displacement has been accompanied with considerable hypertrophy of mucous membrane of the wall where the ovule was grafted, and thus the pedicle was formed.

It is scarcely possible to determine truly the causes which have produced the displacement. It is most probable that it has been induced by uterine contractions, shortly after the affixing of the ovule; the pedicle then was the result of the sudden drawing upon the membrane. The subsequent development in the neck was a true secondary cervical pregnancy, producing a mechanical dilatation of the part.

The displacement was the commencement of an abortion, which would go on gradually, finally ending in hemorrhage.

It also shows by the mortification, that the fœtal envelopes may be perforated and the embryo expelled, while its appendages remain either wholly or partially *in situ*. Then the clots upon such an interrupted pregnancy may finish by forming what is called a fibrous polypus. According to Kiwisch, they are accompanied by profuse metrorrhagia and intense uterine pains for a variable time. He considers this explanation of the formation of such polypi as admissible.—(*Zeitschrift der Gesellschaft der Aerzte zu Wien*, No. 33, 1860; *Gaz. Hebdom.*)

XI.—*Intra-Uterine Small-pox.* By DR. ALEXANDER R. SIMPSON, of Edinburgh.

Mrs. McL., aged twenty-three, pregnant seven months, was attacked with small-pox, February seventh, in the discrete form, which ran its course as usual, and by the eighteenth day she was able to go out. Up to this time the fœtal movements were active, but on the twenty-sixth they became feebler and less frequent. By March seventh they had entirely ceased. The preceding day, when walking, she suddenly felt a weight or falling in the womb. On the twenty-second she was delivered of a dead fœtus, presenting evidences of having suffered with variola. The attack seems to have been mild, and the death to have occurred from fibrous degeneration of the placenta. The case, however, is very interesting, as showing exactly the date of the onset of the disease in the fœtus. The mother suffered, and after her recovery the fœtus was attacked.

*Ibid.* By DR. BRUCE, of Edinburgh.

This instance, as was the case with the other, was brought to the attention of the Edinburgh Obstetrical Society.

Mrs. G. passed favorably through a modified attack of small-pox. She was considerably advanced in pregnancy, having quickened some time previously. About two weeks after, the fœtal heart could not be heard. From this circumstance, and her sense of weight in the lower part of the abdomen, etc., it was concluded that the fœtus was dead, and premature labor was anticipated. About one month later she was delivered of a fœtus of six months, which had evidently been dead for some time. On examination of the body, marks of small-pox were found in various places. It would appear that its death occurred at the time when the mother was convalescing from the disease.

In the debate which ensued, other cases of intra-uterine disease were mentioned, together with instances where, though the mother had suffered severely, no traces of the disease could be found upon the child.—(*Edinburgh Medical Journal*, November, 1860.)

